# Study protocol for a randomized controlled trial: evaluating the effect of isokinetic eccentric training of the hamstring on knee function and walking function after total knee arthroplasty

**DOI:** 10.3389/fmed.2024.1404736

**Published:** 2024-05-23

**Authors:** Tianjun Zhai, Yongjia Song, Jianqing Su, Ruiren Wu, Jie Wang, Zengqiao Zhang, Wei Feng

**Affiliations:** ^1^School of Rehabilitation Science, Shanghai University of Traditional Chinese Medicine, Shanghai, China; ^2^Rehabilitation Department, The Second Rehabilitation Hospital of Shanghai, Shanghai, China; ^3^Tuina Department, Yueyang Hospital of Integrated Traditional Chinese and Western Medicine Affiliated to Shanghai University of Traditional Chinese Medicine, Shanghai, China

**Keywords:** rehabilitation, TKA, postoperative training, RCT, intervention

## Abstract

**Introduction:**

Total knee arthroplasty (TKA) is a widely-used treatment for end-stage knee osteoarthritis. However, it is common for patients to experience issues with knee joint function and abnormal gait following the surgery. Previous studies have primarily focused on concentric contraction of the quadriceps during TKA, neglecting the potential benefits of eccentric isokinetic training for the hamstrings. This protocol outlines a randomized, single-blind, controlled trial aimed at assessing the impact of eccentric isokinetic training for the hamstring muscles on pain, function, and gait in patients after TKA.

**Methods and analysis:**

Fifty participants between the ages of 50 and 80 with knee osteoarthritis undergo unilateral total knee arthroplasty (TKA) for the first time. They will be transferred to the rehabilitation department 10–14 days after the operation. The participants are randomly divided into two groups, with 25 participants in each group: the control group and the Hamstring training group. The Control group will receive routine rehabilitation treatment, while the Hamstring training group will receive a combination of routine rehabilitation treatment and isokinetic eccentric training of the hamstring. The intervention will last four consecutive weeks. Both groups will be assessed at three different times: before the intervention, after 4 weeks of intervention, and 4 weeks after the interventions (follow-up). The primary outcome will be functional capacity (TUGT) and Hospital for Special knee Score (HSS). Secondary outcomes will be knee-related health status (isokinetic knee position perception, Peak torque of hamstring strength), pain intensity (Visual analog scale, VAS) and 3D gait analysis.

**Discussion:**

The study aims to provide relevant evidence on the effectiveness of eccentric hamstring muscle contraction training in improving knee joint function and walking function after TKA.

**Clinical trial registration:**

https://www.chictr.org.cn/showproj.html?proj=195544, Identifier ChiCTR2300073497.

## Introduction

Knee osteoarthritis is a chronic joint disease mainly occurring in middle-aged and older adults. The incidence rate of knee osteoarthritis is increasing due to the aging of the population, which is causing a significant economic burden on society (1, 2). Total Knee Arthroplasty (TKA) is a prevalent intervention for managing end-stage knee osteoarthritis, primarily in the aging population, which significantly alleviates pain and improves joint function (3). Despite the surgery’s effectiveness, many patients post-TKA experience persistent challenges in regaining optimal knee function and a normal gait pattern (4). Current rehabilitation protocols predominantly focus on the quadriceps and largely employ concentric muscle training strategies (5, 6). This conventional approach, while beneficial, often fails to address the full spectrum of functional recovery (7), particularly in the eccentric control of the hamstring muscles, which play a crucial role in joint stability and gait dynamics.

The activation pattern of the muscles of the lower extremities, which relies on muscle synergy, changes with age. The activation of hamstring muscle cooperative activity in older adults over 60 is about 1.6 times that of young people during daily activities. The activity of muscles around the ankle joint in these individuals is also relatively higher (8). As a result, the importance of hamstrings in human sports increases with age. For the functional recovery of early patients after TKA, the increase in hamstring activity is a natural choice of the body. It can stabilize the knee joint by increasing pressure (9), improve eccentric control of the load, and increase the protection of ligaments around the knee joint during standing phase (10). It is a natural compensatory adaptation of the body to weakness, pain, and local environmental changes in the quadriceps femoris (11). This is not a “bad adaptation,” but the body’s coping mechanism for maintaining function and managing pain.

Concentric contraction is used primarily in hamstring muscle strength training rather than eccentric contraction. However, compared to concentric contraction training, Eccentric training is known for its superior efficacy in enhancing muscle strength, improving joint stability, and increasing the resilience of muscle tendons (12). This form of training could potentially offer substantial improvements in the functional recovery phases following TKA. However, the specific benefits of eccentric hamstring training in post-TKA rehabilitation remain underexplored and poorly documented in the existing literature.

Our study aims to bridge this gap by conducting a comprehensive analysis of isokinetic eccentric training for the hamstrings on knee function and gait rehabilitation post-TKA. We hypothesize that incorporating eccentric hamstring exercises into the postoperative rehabilitation protocol will result in better functional outcomes compared to traditional methods focusing solely on the quadriceps. This study aims to furnish concrete proof that endorses a comprehensive strategy for muscle training during TKA recovery, which could transform existing rehabilitation methodologies by prioritizing the equilibrium of muscle forces and the refinement of joint mechanics.

## Methods

### Aims

The study will explore the effect of hamstring isokinetic eccentric training on early lower limb motor function recovery in patients after TKA. Mainly includes: Comparing the effects of hamstring isokinetic eccentric contraction training on the affected side hamstring muscle strength, knee joint position sense, knee joint function score and three-dimensional gait in patients with early TKA surgery; analysis and discussion of hamstring isokinetic eccentric contraction training Effects of contraction training on knee joint muscle strength, proprioception and walking function in patients with TKA.

### Trial design

**Figure 1:**
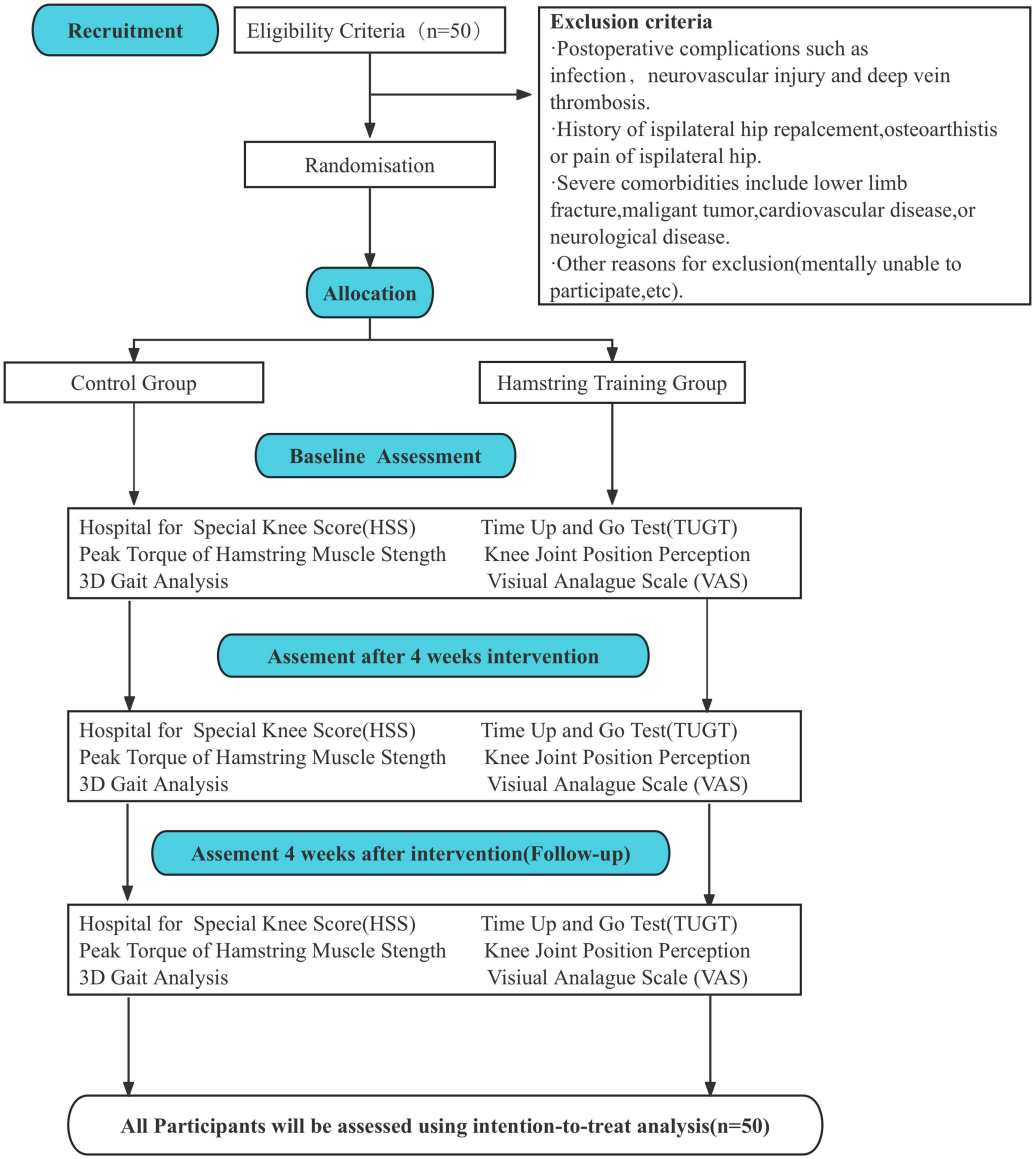
Design and flow of participants through the trial.

This study is a prospective randomized controlled trial. The study will be developed in Four stages, according to Figure 1. The eligible participants will be recruited from The Second Rehabilitation Hospital of Shanghai. The doctor in the rehabilitation medicine department of the hospital informed the eligible patients of the research plan. All participants provided their written informed consent. Baseline evaluation will include assessment of name, sex, height, weight, age, and affected limb. After random assignment to different intervention groups, participants will enter the intervention phase. This phase will consist of two different intervention programs lasting 4 weeks. The outcome indexes will be evaluated before intervention, after 4-Week intervention and after 4 weeks follow-up. This study follows the CONSORT statement (13) and the ethical principles of the Declaration of Helsinki.

### Participants and setting

Individuals after TKA will be recruited between August 2023 and August 2025 at Shanghai second Rehabilitation Hospital. At least fifty participants will be included in this study.

### Inclusion criteria

- Were aged between 50 to 80 after TKA.
- Had knee osteoarthritis and received unilateral TKA for the first time and was transferred to the rehabilitation department 10–14 days after the operation.
- During the 10–14 days postoperative period, participants did not engage in strength training related to the quadriceps and hamstrings.
- Were operated with the same type of posterior stabilized prosthesis;
- Were able to cooperate with rehabilitation training.
- Had no regular exercise habits.
- Provided written informed consent.

### Exclusion criteria

- Postoperative complications include infection, neurovascular injury, or symptomatic deep vein thrombosis.
- History of ipsilateral hip replacement, osteoarthritis, or pain of ipsilateral hip.
- Severe comorbidities include lower limb fracture, malignant tumor, cardiovascular disease, or neurological disease.
- Other reasons for exclusion (mentally unable to participate, etc.).

After randomization, the criterion for dropping out will be the participants not attending the assessments (4 weeks, and follow-ups).

### Sample size

Based on the work being done by the team when formulating the plan, we estimated that the difference in TUGT time between the two groups was 1.87 s. With a 2-sided α error of 0.05 and a β error of 0.20, the sample size is estimated at 22 patients in each group. Due to a dropout rate of about 10%, 50 patients are needed.

### Recruitment procedure

Patients who meet the inclusion criteria and are receiving treatment at The Second Rehabilitation Hospital of Shanghai will be invited to participate in the RCT study. To ensure maximum recruitment, doctors, evaluators, and physiotherapists involved in the recruitment process have received training and instructions. The recruiting doctor will provide participants with both oral and written information about the study. If the participant or their guardian agrees to participate in the study, they will be required to sign a written informed consent.

### Randomization procedure and concealment of allocation

Patients who meet the inclusion criteria and are willing to participate will be randomized in a 1:1 allocation ratio after the baseline assessment. SPSS25.0 (IBM Corp., Armonk, NY, United States) statistical software was used to generate random serial numbers. These serial numbers were then printed on cards, sealed in paper envelopes, and the same serial number was written on the outside of each envelope. Patients opened the envelopes in the order of their recruitment. The participants will be randomly divided into two groups: the experimental group (hamstring training group) consisting of 25 cases, and the control group (routine rehabilitation group) consisting of 25 cases. The allocation numbers were concealed in opaque sealed envelopes prepared by a central study coordinator. These envelopes will only be accessible to the central study coordinator, who will open them after obtaining informed consent and baseline measures.

### Blinding

This study used the single-blind principle. The investigator responsible for the patient assignment was uninvolved in-patient recruitment, evaluation, treatment, and data statistics. The study evaluators were unaware of the allocation of patients. No blinding could be performed by the physical therapy staff or the patients.

### Rehabilitation interventions

Patients will be randomized to one of two treatments initiated as soon as possible after randomization. Both groups of patients received routine rehabilitation treatment, including manual therapy, quadriceps femoris, calf muscle strength training, knee joint flexibility and range of motion training, walking, and stair climbing (14). 40 minutes per day, five days per week, for a total of four weeks.

### Control group

The patients in the control group were given routine rehabilitation treatment:

- Body position: the affected knee was kept straight.
- Isometric contraction training of the anterior and posterior muscle groups of the knee joint: 10 s contraction, 10 s relaxation, 10 times per group, three groups per day.
- Ankle pump training: 10 times per group, three groups per day.
- Knee flexion and extension training: 10 times per group, three groups per day.
- Straight leg lifting training: 10 times per group and three groups per day.
- Hip abduction training inside lying position: adopt a healthy side-lying position, and slowly put down the affected hip after 5 ~ 10 s of abduction.
- Patellofemoral joint mobilization: the knee joint being fully extended with the leg muscles relaxed, the patella being loosened up, down, left, and right. These exercises were repeated 30 times for each group, and each direction could last about 10 s.
- Knee joint range of motion training: with the patient in the supine position, the healthy side of the lower limb was bent, the affected side of the lower limb was first relaxed and straightened, and the patient was then required to slide back, so that the knee joint could be flexed to an acceptable angle for 5 s, and then straightened 10 times per group, and three groups per day.
- Walking and stair climbing training: the walking distance and speed up and down stairs were gradually increased.
- Balance and proprioception training: the patient stands on the soft or hemispheric plane with eyes open or closed and maintains body balance for 30 s.

### Hamstring training group

The patients in the hamstring training group received routine rehabilitation treatment. Moreover, isokinetic eccentric training of the hamstring muscle was simultaneously added.

- Preparatory activities before training: the hamstring muscle of the affected side of the patient should be relaxed and massaged for 5 min, and the patient should be guided to perform stretching exercises of the hamstring muscle to prevent discomfort during training.
- Isokinetic eccentric contraction training of hamstrings: Using the Biodex 4 isokinetic muscle strength training system (BIODEX, United States), the patient performed eccentric isokinetic training of hamstring muscle in the range of 20 ° ~ 80 ° of knee flexion on the affected side (15). The angular velocity was selected as 30°/s and 60°/s. Isokinetic eccentric hamstrings training was performed 10 times in each group, three training groups with an interval of 1 min. No active muscle movement was performed during eccentric knee joint reduction. Training should be terminated if the patient experiences discomfort or pain due to eccentric training.
- Relaxation activity after training: the hamstrings should be massaged and relaxed for 5 min after training.

### Study outcomes

The patients will be evaluated before rehabilitation training, after four weeks of training and four weeks after intervention (follow-up). Each evaluation will be performed by the same trained doctor for a comprehensive clinical examination and evaluation of outcome indicators. The evaluators will be blinded in this study and will be uninformed about the randomization of the participants or has the opportunity to participate in the research intervention. Adverse events will be recorded throughout the study.

### Baseline characteristics

Patient characteristics, including gender, height, weight, age, and affected limb, will be collected for subsequent baseline analysis.

### Primary outcome

The primary outcomes measured in this study are Timed Up and Go Test (TUGT) and Hospital for special surgery knee score (HSS).

#### Timed up and go test

TUGT test primarily assesses the individual’s mobility, balance, neuromuscular function, and flexibility. It is common for individuals to experience a reduction in lower limb strength, gait, balance, mobility, and body coordination After TKA, these factors can influence the outcomes of the TUGT test (16). For the experiment, a chair with armrests is prepared and a stopwatch is used. A colored tape is placed 3 meters in front of the chair to serve as a mark line. The participant is instructed to sit upright on the chair, with their back against the backrest and hands on the armrests. Upon hearing the command ‘start’, the participant stands up from the chair and walk towards it as quickly and safely as possible. They walk forward for 3 meters until reaching the marked line, then turn around and return to the chair to sit down. The total time takes for this task will be recorded in seconds. Notes: (1) Participant should wear comfortable clothing and flat shoes during the test. (2) The watch should be started when the participant ‘s back leaves the back of the chair, and stopped when the participant sits down in an upright posture with their back touching the back of the chair. (3) Sufficient time should be given to the subjects to adapt and practice before starting the test, ensuring their understanding of the entire test content. (4) The rater should refrain from offering any verbal encouragement or physical assistance to the participant, but should remain nearby to ensure their safety.

#### Hospital for special surgery knee score (HSS)

In 1976, the Hospital for Special Surgery in the United States proposed this 100-point scoring system. It has been found to have high accuracy in assessing the recovery of joint function after TKA surgery and comparing the pre-and post-surgery outcomes. It is particularly effective in evaluating the motion of both the patellofemoral and femoro-tibial joints, especially in the immediate post-surgery period. HSS is used to assess knee joint function after surgery, mainly including pain, joint range of motion, function, muscle strength, knee joint flexion deformity, and knee joint instability (17). Recovery of the knee joint of the participant is judged by the score, the higher the score, the better.

### Secondary outcome

The secondary outcomes measured in this study are Peak torque of hamstring muscle strength, Knee joint position perception, 3D (Three-dimensional) gait analysis and VAS.

#### Peak torque of hamstring muscle strength

The BIODEX isokinetic muscle strength training system is used to test the maximum torque of the muscle strength (18) third times: before intervention, after intervention and after 4 weeks’ follow-up.

Test method:(1) Participant sits on the isokinetic dynamometer chair. To prevent unnecessary movements, the shoulders, chest, and hips were securely strapped. The cuff of the dynamometer arm was attached near the ankle on the same side. (2) The back seat of the dynamometer was tilted at an angle of 75–85° backward. (3) The angle on the dynamometer was set from 0° (indicating full knee extension) to 90° knee flexion, with a speed of 90°/s. Prior to taking the baseline measurements, each participant performed the movement three times with submaximal effort for practice.

Subsequently, participant is instructed to perform the knee bend movement with maximum effort. Three measurements are taken, and the highest reading obtained is used for data analysis.

#### Knee joint position perception

Using the evaluation software in the isokinetic muscle strength test training system, the participants are evaluated third times: before intervention, after intervention and after 4 weeks’ follow-up.

The test method involved the following steps: (1) Participant sits on the seat with their hips and knees bent at 90 degrees. The participant’s shoulders, waist, and thighs are fixed using shoulder, waist, and thigh fixing belts. (2) The test and evaluation system is calibrated, and the knee joint proprioception test is selected. Two target angles are chosen for the knee joint to flex 30 degrees. The angular velocity of the power head of the constant velocity instrument is set at 1°/s. Each test evaluation is repeated 3 times with a 10 s interval between tests. (3) To avoid visual interference during the test, the participant wears goggles. The participant also holds the electric button. The evaluator instructs the participant to passively raise their calf to the target angle and hold it for 10 s. The participant should remember the current position carefully. After resetting, the instrument will lift the participant’s lower limbs while rotating. When the participant feels that the knee joint angle has reached the target position, they should press the switch. At this point, the instrument stops rotating and records the error between the actual angle and the target angle (19).

Each target angle should be tested 3 times and then the average value is calculated. A smaller recorded error indicates good proprioception. Participants are allowed to familiarize themselves with the procedure once before each test.

#### The 3D gait analysis

The 3D gait analysis is evaluated in the 3D Gait Laboratory of the Rehabilitation Medicine Department of the Second Rehabilitation Hospital of Shanghai. The test will be administered by experienced therapists who are proficient in the steps. During the laboratory session of gait analysis, the patients are instructed to wear tight clothing and walk comfortably.

Test method:(1) Before conducting the experiment, it is important to close all doors and windows and tightly close the curtains to prevent any outside light from entering. In preparation for the experiment, the participants are instructed to remove their shoes and socks and wear tights. (2) The tester then place a reflective marker ball, measuring 14 mm in diameter, on the participant. A total of 48 marker points were selected based on the human body model for both dynamic and static data collections. These marker balls are attached to specific locations on the participant’s body, such as the anterior superior iliac spine, posterior superior iliac spine, lower lateral third of the thigh, lateral condyle of the knee joint, lower third of the lateral manubrium, top of the lateral malleolus, second metatarsal head, and heel. (3) The participants are instructed to walk on a 10-meter-long flat ground at a natural speed. This is repeated three times in total. Kinematic analysis will be conducted for the entire gait cycle, starting from the third trial. Three-dimensional gait quantitative parameters, including spatiotemporal parameters such as pace, stride length, stride frequency, support phase, and knee joint angle, will be recorded and analyzed (20). The data will be analyzed using appropriate software. The participants will be evaluated third times: before intervention, after intervention and after 4 weeks follow-up.

#### VAS (visual analogue scale)

VAS is a widely used pain scoring standard (21). It consists of a 10-point scale, with 0 indicating no pain and 10 indicating severe pain. The intermediate points on the scale represent varying degrees of pain. Patients are asked to mark a point on a horizontal line to indicate their level of pain. Mild pain is represented by 1–3 points, moderate pain by 4–6 points, and severe pain by 7–10 points. Assessing the pain level before and after hamstring training can provide insights into the impact of pain on patients’ gait.

#### Minimizing missing data

In order to avoid and minimize missing data, all participants will receive face-to-face education sessions conducted by qualified doctors upon enrollment. These sessions will cover various topics including potential outcomes after TKA, pain management, and the significance of physical therapy on recovery. Additionally, participants will be informed about potential complications that may arise during the intervention. Regular phone contact will serve as a reminder for participants to stay connected with the study and its evaluation. At 4 weeks’ follow-up, researchers will contact participants via phone and request their presence at the hospital for relevant assessments.

#### Data management

**Figure 2:**
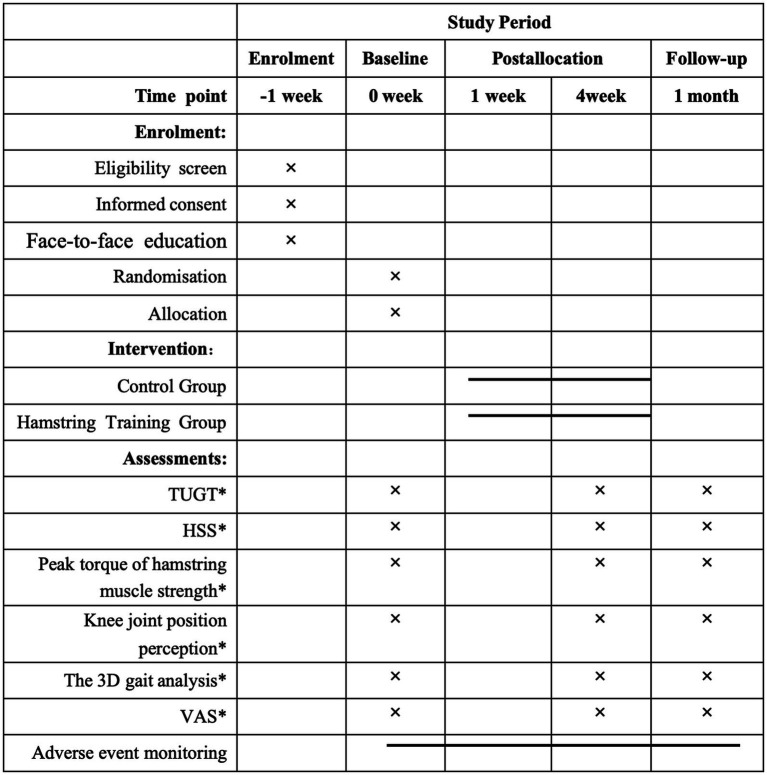
Schedule of enrolment, intervention and assessment.

The data will be collected in the Rehabilitation Department of the Second Rehabilitation Hospital of Shanghai and stored in password-protected computer files. Access to these files will be restricted to researchers only. The principal investigator will also maintain a backup copy of all the information. The research findings will be disseminated through related papers. Please refer to Figure 2 for an overview of collection of the different outcomes.

#### Data monitoring

A supervisor professor among the researchers will closely monitor the participants’ performance throughout the study, including assessment and intervention phases. The supervisor will be unbiased and blind to the group assignments. They will also oversee database management and conduct statistical analysis, while keeping an eye out for any potential adverse events.

Any adverse events that occur during assessment, intervention, or follow-up will be carefully recorded and reported. These adverse events may include symptoms such as increased pain or local redness. The monitoring professor will also ensure the integrity of the data, and The Second Rehabilitation Hospital of Shanghai Data Monitoring Committee will have access to patient assignments.

#### Harm

The study will meticulously document any relevant changes in participant data. In the event of adverse reactions or complications during treatment, these will be reported alongside other trial results.

#### Statistical analysis

SPSS24.0 statistical software was used for the statistical analysis of the data in this study. Data were presented as mean (SD) or median [Q1–Q3] for quantitative variables and frequency (%) for categorical variables. The design includes two groups and three time points. The Shapiro–Wilk test is used to check the normality of the measurement data; if the data do not follow a normal distribution, transformations will be applied. A two-way ANOVA is conducted to determine whether there is an interaction between group and time factors. If an interaction is detected, we will assess whether time or group has an independent effect. If no interaction is detected, we will determine if there are any main effects. Post-hoc comparisons between groups at the same time points are adjusted using Bonferroni correction to ensure the overall type I error rate for each ANOVA does not exceed 0.05. Minimal clinically significant differences (MCID) were applied to compare the present results. The analyzes were conducted intention-to-treat by an independent statistician blinded to group allocation. The significance level is set at α = 0.05.

#### Publication

The research findings will be shared in scientific journals, making them accessible to the orthopedics and rehabilitation community. Additionally, these findings will be discussed and debated at scientific conferences to encourage meaningful discourse.

## Discussion

The hamstrings are muscles that span both joints and play a role in knee flexion and hip extension when exerting force. As antagonists, the hamstrings and quadriceps work together to maintain knee joint stability. During walking, the eccentric contraction of the hamstrings can slow down the swing of the calf, preventing knee joint instability (22). Therefore, in early postoperative treatment, it is important to not only focus on strengthening the quadriceps, but also improve the muscle balance of the knee joint and exercise the hamstrings. Balanced muscle strength is crucial for smooth completion of human body movements. However, the quadriceps are naturally stronger than the hamstrings in the knee joint. If solely focus on quadriceps strength training, it can lead to an imbalance in muscle strength of the knee joint (23). To address this, it is necessary to also strengthen the hamstring muscles which is often overlooked in clinical rehabilitation and further worsen the problem. Study have shown that the older adults exhibits reduced quadriceps activation and increased hamstring activity compared to healthy young individuals of the same age and gender (24). This highlights the need to pay more attention to the impact of hamstring strength on knee joint stability in older adults patients. Insufficient hamstring strength can negatively affect the stability of the knee joint. For instance, during the concentric contraction of the quadriceps or the stretching of the lower limbs, the hamstrings contract eccentrically. If the hamstrings lack adequate eccentric control ability, they are unable to effectively stabilize the knee joint (25).

Conventional rehabilitation training typically focuses on concentric contraction resistance in muscle strength training. Previous research has indicated that eccentric contraction resistance can effectively enhance the strength of target muscles, sometimes even increasing strength by 30–40% (26). Studies have also found that eccentric training has benefits in increasing the cross-sectional area of muscles, promoting muscle fiber synthesis, and improving muscle strength (24). Additionally, a study by LaStayo discovered that cardiopulmonary oxygen consumption during eccentric contraction resistance training was lower (27). This implies that while eccentric exercise reduces cardiopulmonary oxygen demand by 20%, muscle strength increases rapidly due to improved metabolic efficiency. Therefore, under the same metabolic workload, eccentric contraction training can enhance muscle strength to a greater extent (28). A systematic review analysis conducted by Roig further supports the effectiveness of muscle eccentric contraction resistance exercise in increasing strength compared to normal concentric contraction resistance exercise. Roig also suggests that due to its ability to enhance muscle strength with minimal energy expenditure, eccentric contraction training may be more suitable for older adults patients with low exercise tolerance (29). Considering that most individuals undergoing TKA surgery are middle-aged and older adults patients, eccentric contraction resistance training may be more appropriate. Unlike traditional hamstring muscle training methods that involve the use of elastic bands and manual resistance training, this study incorporates the use of isokinetic strength training equipment. Postoperative hamstring training has historically been relatively simple, but it poses challenges in terms of training intensity control and risk of injury for patients. In recent years, with advancements in medical equipment, isokinetic muscle strength training equipment has been increasingly utilized in rehabilitation training.

Numerous studies have examined post-TKA rehabilitation and hamstring muscle training. However, this program represents the first study to investigate the application of hamstring muscle isokinetic eccentric contraction training specifically for TKA patients. The participants are divided into two intervention groups: one receiving routine physical therapy and the other undergoing hamstring isokinetic eccentric contractions. The purpose of this study is to assess walking function, knee function, and pain severity in patients with TKA. TUGT test and HSS primarily assesses the individual’s mobility, balance, neuromuscular function, and flexibility, which can reflect the participants’ walking function and knee joint function. To provide an objective evaluation, three-dimensional gait analysis technology was utilized, which is considered the ‘gold standard’ for analyzing gait problems. The use of functional 3D gait analysis will help to know whether the hamstring isokinetic eccentric exercise will influence on walking function after TKA or not. This analysis system allows for a more precise assessment of the effects of hamstring muscle isokinetic eccentric contraction on TKA patients’ walking function. Peak torque refers to the maximum torque produced when the muscle is contracted with maximum force. In this study, the BIODEX isokinetic muscle strength test training system will be used to assess the Peak torque of hamstring muscle strength before and after the treatment. The aim is to determine whether isokinetic eccentric exercise could increase hamstring muscle strength. The Knee joint position perception test is to judge whether there is improvement through its position sense. One aims of this project is to know whether the hamstring isokinetic eccentric exercise will influence on walking function after TKA or not. Thus Three-dimensional gait analysis is a key component of this study and will provide important results. Additionally, VAS was used to compare any changes in pain levels after the isokinetic eccentric training of hamstring muscles.

This study has certain limitations, including just a single center and lack of long-term follow-up. While the characteristics of intervention measures may not allow for therapist blinding, evaluator and data analyst blinding will be implemented to reduce bias as much as possible. To enhance the credibility of the conclusions presented in this paper, future research should aim to multi-center research and extend the follow-up period. This will provide further clarification on the clinical effectiveness of hamstring muscle isokinetic eccentric contraction training.

## Author’s note

The study results will be released in conferences, such as scientific conferences, internationally and nationally, and through articles published in peer-reviewed journals. The results will be frequently presented to the supervising professor (WF).

## Ethics statement

The studies involving humans were approved by the Ethics Committee of the Second Rehabilitation Hospital of Shanghai. The studies were conducted in accordance with the local legislation and institutional requirements. The participants provided their written informed consent to participate in this study.

## Author contributions

TZ: Methodology, Investigation, Funding acquisition, Data curation, Writing – review & editing, Writing – original draft. YS: Writing – review & editing, Methodology, Data curation. JS: Writing – review & editing, Validation, Software, Project administration, Methodology. RW: Writing – review & editing, Resources, Project administration, Investigation, Data curation. JW: Writing – review & editing, Visualization, Validation, Software, Project Administration, Investigation. ZZ: Writing – review & editing, Validation, Resources, Methodology, Formal analysis, Data curation. WF: Writing – review & editing, Supervision, Conceptualization.
